# Snake Venom Compounds: A New Frontier in the Battle Against Antibiotic-Resistant Infections

**DOI:** 10.3390/toxins17050221

**Published:** 2025-05-01

**Authors:** Barathan Muttiah, Alfizah Hanafiah

**Affiliations:** 1 Department of Medical Microbiology and Immunology, Faculty of Medicine, Universiti Kebangsaan Malaysia, Cheras, Kuala Lumpur 56000, Malaysia; 2 GUT Research Group, Universiti Kebangsaan Malaysia, Kuala Lumpur 56000, Malaysia

**Keywords:** snake venom, antibiotic resistance, venom components, antibacterial agent, alternative therapies

## Abstract

The occurrence of antibiotic-resistant bacteria is a serious global health issue, and it emphasizes the need for novel antimicrobial agents. This review explores the potential of snake venom as another alternative strategy against antimicrobial resistance. Snake venoms are complex combinations of bioactive peptides and proteins, including metalloproteases (MPs), serine proteases (SPs), phospholipase A_2_ (PLA_2_) enzymes, three-finger toxins (3FTXs), cysteine-rich secretory proteins (CRISPs), L-amino acid oxidases (LAAOs), and antimicrobial peptides (AMPs). The antibacterial products possess wide-spectrum antibacterial activity against resistant microbes via diverse mechanisms such as cell membrane disruption, enzymatic hydrolysis of microbial structures, generation of oxidative stress, inhibition of biofilms, and immunomodulation. Strong antimicrobial activity is reported by most studies, but these are mostly restricted to in vitro testing with low translational use. Although preliminary insights into molecular targets and physiological effects exist, further studies are needed to clarify long-term safety and therapeutic potential. Special attention is given to snake venom-derived extracellular vesicles (SVEVs), which enhance the therapeutic potential of venom toxins by protecting them from degradation, improving bioavailability, and facilitating targeted delivery. Furthermore, innovative delivery strategies such as PEGylation, liposomes, hydrogels, microneedle patches, biopolymer films, and nanoparticles are discussed for their role in reducing systemic toxicity and enhancing antimicrobial efficacy. The rational modification of venom-derived peptides further expands their therapeutic utility by improving pharmacokinetics and minimizing off-target effects. Together, these approaches highlight the translational potential of snake venom-based therapies as next-generation antimicrobials in the fight against resistant infections. By outlining these challenges and directions, this review positions snake venom as an overlooked but fertile resource in the battle against antibiotic resistance.

## 1. Introduction

The rising incidence of antibiotic-resistant bacteria is a significant global health issue, highlighting the dire necessity of searching for new antimicrobial agents [1]. The World Health Organization has listed 24 bacterial pathogens as priorities, and most of them have been found to be resistant to last-resort antibiotics, thereby posing a critical threat to public health [2]. Multidrug-resistant (MDR) pathogens, including *Acinetobacter baumannii*, *Pseudomonas aeruginosa*, and *Staphylococcus aureus*, have been associated with high morbidity and mortality rates, especially in the healthcare environment [3]. The development and dissemination of resistance are fueled by a series of factors that span from the excessive use of antibiotics to inadequate sanitation practices, environmental contamination, and genetic adaptation via horizontal gene transfer and selective pressures [4]. To thwart these issues, new treatment strategies are being investigated—such as nanotechnology, bacteriophage therapy [5], CRISPR-mediated gene editing [6], and antimicrobial peptides [7]—to combat the drawbacks associated with conventional antibiotics.

Among the various emerging strategies to combat antimicrobial resistance, snake venom has garnered growing attention due to its diverse array of bioactive proteins and peptides with potent antimicrobial properties [8]. Snake venoms are complex mixtures comprising enzymes, neurotoxins, phospholipases, and metalloproteinases, components traditionally studied for their toxic effects but increasingly recognized for their therapeutic potential [9]. Notably, venom compositions vary significantly across species, influenced by genetic and ecological factors such as evolutionary lineage, gene expression patterns, geographic distribution, habitat type, prey availability, and environmental pressures [10]. In general, elapid venoms tend to be neurotoxic, whereas viperid venoms exhibit hemotoxic activity [7]. Specific venom-derived molecules, such as phospholipases A_2_, snaclecs, and serine protease inhibitors, have demonstrated antibacterial effects through mechanisms including membrane disruption, toxin neutralization, and biofilm inhibition [8]. Venom-based peptides are also under investigation for their anticancer, anticoagulant, and neuroprotective properties [11]. These findings underscore the pharmacological versatility of venom constituents and reinforce their potential specifically as novel antimicrobial agents, especially in the fight against MDR pathogens [12].

While several recent reviews have addressed the antimicrobial effects of snake venom [13,14,15,16,17], our review will further evaluate the antimicrobial potential of snake venom constituents and identify novel delivery strategies—both nanoparticle-free and nanoparticle-based, such as exosomes, nanoencapsulation, PEGylation, and non-nanoparticle systems like liposomes, hydrogels, microneedle patches, and biopolymer films—to enhance their translational utility in the treatment of antibiotic-resistant bacteria and overcome current limitations and enable their effective application against MDR bacteria. These integrative approaches which remain underexplored in the literature may represent the next frontier in snake venom-based antimicrobial therapy.

## 2. Composition of Snake Venom and Antibacterial Constituents

Snake venom is a highly complex biochemical cocktail composed predominantly of proteins that have evolved to subdue prey and initiate digestion [8]. However, beyond their traditional roles in envenomation, these venom components have recently attracted attention for their potential biomedical applications [11], particularly in combating bacterial infections [12]. The rise of antibiotic resistance has necessitated the search for alternative antimicrobial agents, and snake venom proteins exhibit potent antibacterial properties that may serve as the foundation for novel therapeutic strategies [17]. This section explores the major protein families in snake venom that demonstrate antibacterial activities, focusing on their structure, mechanisms of action, and potential applications.

### 2.1. Metalloproteases (MPs)

Snake venom metalloproteases (MPs) are among the most abundant components in viper and pit viper venoms, constituting 20–60% of the total venom protein content [18]. These enzymes are categorized into three primary classes—P-I, P-II, and P-III—based on their structural complexity and domain organization [19]. Their primary function in envenomation is to degrade extracellular matrix proteins, leading to tissue degradation, hemorrhage, and inflammation during envenomation [20], but they also display remarkable antibacterial properties [21]. Structurally, MPs possess a modular design that includes a metalloprotease domain responsible for proteolysis, a disintegrin-like domain, and a cysteine-rich domain [22]. Their enzymatic activity is dependent on metal ions, with zinc acting as a catalytic cofactor and calcium contributing to structural stability [19]. The antibacterial effects of MPs stem from their ability to disrupt bacterial membranes, inhibit ion channels crucial for bacterial homeostasis, and act against both Gram-positive and Gram-negative bacteria [23]. An MP from Chinese viper *Agkistrodon halys* has been found to damage the membrane integrity of MDR bacterial strains such as *Proteus vulgaris*, along with other bacteria like *S. aureus* and *Burkholderia pseudomallei* [17]. *Agkistrodon halys* metalloproteinase (AHM) has also demonstrated effectiveness against *Proteus mirabilis* [21] whereby variants of AHM that were highly effective (minimum inhibitory concentration (MIC): 1.875–60 μM) were also non-toxic to human monocytic cells (U-937) up to 1 mm. Similarly, PIII-MP (73/60 kDa) isolated from *Bothriopsis oligolepis* has demonstrated potent activity against *S. aureus* [24]. These enzymes exhibit low MICs, suggesting high efficacy even at minimal doses, reinforcing their potential for development into antibacterial agents. The viper metalloproteinase from *Agkistrodon halys pallas* and proteases from *Bothrops arietans* all display strong antimicrobial and anti-biofilm properties. Mechanistically, these effects may stem from the hydrolysis of essential bacterial surface proteins or the destabilization of membranes, similar to their action on mammalian extracellular matrix components. The structural resemblance of snake venom MPs to mammalian matrix metalloproteinases and ADAM (a disintegrin and metalloproteinase) proteins suggests a conserved substrate recognition mechanism that could extend to microbial targets [25]. While their promising activity against antibiotic-resistant pathogens highlights their therapeutic potential, further studies are necessary to fully characterize their specificity, toxicity, stability, and pharmacokinetics in physiological conditions to support clinical translation.

### 2.2. Serine Proteases (SPs)

Serine proteases (SPs), also known as snake venom thrombin-like enzymes (TLEs), are single-chain enzymes belonging to the S1 family of chymotrypsins, with molecular weights ranging from 26 to 33 kDa [26]. Despite their high sequence identity (51–98%), they exhibit remarkable substrate specificity and serve multiple functions, including blood coagulation and fibrinolysis [27,28]. Some function as TLEs, while others act as kallikrein-like enzymes, inducing vasodilation and hypotension [29]. Their antibacterial mechanisms involve direct membrane disruption, proteolytic degradation of essential bacterial proteins, and broad-spectrum effects against bacteria, fungi, and viruses. SPs have not been associated with antimicrobial activity; however, a specific purified SVSP from *B. oligolepis* venom demonstrated antibacterial activity against *S. aureus* with an MIC of 80 µg/mL against *S. aureus* ATCC 25923 (G+), suggesting a potential for further exploration of SPs for antimicrobial applications [30]. Although their antibacterial potential is significant, challenges such as potential toxicity and incomplete mechanistic understanding need to be addressed before they can be developed for clinical applications.

### 2.3. Phospholipase A_2_s (PLA_2_s)

PLA_2_s are highly abundant in snake venoms and catalyze the hydrolysis of phospholipids, leading to the release of fatty acids and lysophospholipids [31]. They are involved in diverse toxicological and pharmacological effects, including neurotoxicity, myotoxicity, and hemolysis [32]. Structurally, they possess a conserved calcium-binding loop crucial for catalysis and exist in monomeric, dimeric, or oligomeric states [33]. Snake venom phospholipase A_2_s (PLA_2_s), encompassing both acidic (Asp49) and basic (Lys49) isoforms, exhibit significant antibacterial activity, predominantly against Gram-positive bacteria, with some also demonstrating efficacy against Gram-negative strains [34]. Their antimicrobial mechanism is primarily attributed to membrane disruption through enzymatic phospholipid hydrolysis, leading to increased permeability and eventual cell lysis; however, non-catalytic mechanisms have also been documented [14].

Basic PLA_2_ from *Daboia russelii pulchella* displayed greater inhibitory effects against Staphylococcus aureus and Bacillus subtilis compared to *Escherichia coli* and *P. aeruginosa*, with MICs ranging from ~11.1 to 20 µg/mL [35]. Similarly, acidic PLA_2_ from the Indian cobra (*Naja naja*) was effective against both Gram-positive and Gram-negative bacteria, with antibacterial activity closely tied to its enzymatic function—enzymatic inhibition significantly reduced its efficacy [36]. An acidic PLA_2_ from *Porthidium nasutum* (~15.8 kDa) showed potent activity against *S. aureus* without toxicity in murine cells, whereas its basic PLA_2_ counterpart exhibited slight toxicity [37]. PLA_2_ from *Montivipera bornmuelleri* also demonstrated strong activity against *S. aureus* [38], while PLA_2_ from *Bothrops erythromelas* inhibited *S. aureus* with an IC_50_ of 30.2 µM and it also demonstrated significant anti-biofilm activity against *A. baumannii* (IC_50_ = 1.1 µM) [39]. Importantly, a catalytically inactive Lys49 PLA_2_, named LmutTX, from *Lachesis muta muta* exhibited promising antibacterial effects, particularly against methicillin-resistant *S. aureus* (MRSA), with its derived peptides showing low cytotoxicity [40]. Building on this, synthetic biomimetic peptides modeled on the C-terminal region of Lys49 PLA_2_ have emerged as potent agents against multidrug-resistant bacterial strains, highlighting their potential as novel peptide-based antibiotic alternatives [14].

### 2.4. Three-Finger Toxins (3FTxs)

Three-finger toxins (3FTxs) represent a superfamily of non-enzymatic proteins found in the venom of a large number of snake species, particularly in the elapid family (cobras, mambas, and kraits) and colubrids (mildly venomous and harmless snakes) [41]. The proteins have a characteristic three-loop structure stabilized by four conserved disulfide bonds that constitute the core of the molecule. It is from this feature that these toxins have been designated as three-finger toxins [13]. These toxins exhibit diverse biological functions, including neurotoxicity, cardiotoxicity, and cytotoxicity, but have also been recognized for their antimicrobial properties [13]. Although 3FTxs are not usually regarded as the primary antimicrobial molecules in snake venom, there are some preliminary research results that indicate the possibility that certain forms or fragments of 3FTxs can exhibit antibacterial activity. For an example, cardiotoxin-like basic polypeptides (CLBPs) from cobra venom, for example, A5 from *Naja naja*, have exhibited strong antibacterial activity against Gram-positive bacteria like *Bacillus subtilis* [42]. These toxins, with structural homologies to other 3FTXs, appear to exert antibacterial action by binding to bacterial membranes, likely due to their amphipathic nature (having both hydrophobic and hydrophilic regions that interact with cell membranes). This interaction leads to a disruption in the membrane that ultimately results in the death of the bacterial cell [43]. However, research indicates that 3FTXs like cytotoxin 3, CLBP, and alpha-neurotoxins have proven extremely toxic towards Gram-positive bacteria, and their amphiphilic nature and positive charge facilitate selective targeting of bacterial membranes, making them promising leads for antibiotic development [44]. However, these compounds exhibit no activity against Gram-negative bacteria. A review has demonstrated their effectiveness against *S. aureus* (including MRSA strains) and *E. coli* [13]. 3FTxs also have a limitation, as the structural diversity of outer membranes in Gram-negative bacteria that acts as an impediment towards a variety of antimicrobial molecules [45].

### 2.5. Cysteine-Rich Secretory Proteins (CRISPs)

Cysteine-rich secretory proteins (CRISPs) are single-chain proteins (20–30 kDa) with 16 cysteine residues, contributing to their structural stability [46]. They contain three functional regions: a PR-1 domain, a hinge region, and a cysteine-rich domain (CRD) [47]. These proteins are characterized by a cysteine-rich motif and have been identified in several snake venoms, such as *Crotalus viridis viridis* (rattlesnake) and *Philodryas patagoniensis* [48]. Though their main functions in venom are still under study, some CRISPs have exhibited promising activity against trypanosomatids and were also shown to inhibit smooth muscle contraction and cyclic nucleotide-gated ion channels [49]. Some CRISPs are known to modulate ion channels, which may contribute to their antibacterial effects [50]. For example, Patagonin-CRiSP of *Philodryas patagoniensis* was active against Gram-negative bacteria as well as against filamentous fungi. It had an MIC of 15–7.5 μg/mL (0.6–0.3 µM) and minimum bactericidal concentration (MBC) of 30–15 μg/mL (1.2–0.6 µM) against *P. aeruginosa* and *Penicillium expansum* [51]. However, many CRISPs remain functionally uncharacterized, and further research is required to elucidate their antimicrobial mechanisms and therapeutic potential.

### 2.6. L-Amino Acid Oxidases (LAAOs)

LAAOs are flavoenzymes predominantly found in snake venom, certain fish species, and microorganisms [52]. LAAOs are flavoproteins that catalyze the oxidative deamination of L-amino acids, producing α-keto acids, ammonia, and hydrogen peroxide [53]. Their antibacterial activity is primarily attributed to hydrogen peroxide production, which induces oxidative stress in bacteria, leading to cell membrane damage and biofilm inhibition [54]. For instance, *Ophiophagus hannah* LAAO effectively acts against *S. aureus* and *S. epidermidis*, including MRSA with a lowest MIC value of 7.5 μg/mL, via H_2_O_2_ production [55]. Similarly, *Trimeresurus jerdonii* LAAO has broad-spectrum antibacterial activity against exhibited antibacterial activity against *E. coli*, *S. aureus*, *P. aeruginosa*, and *B. megaterium*, with the activity abolished by catalase (indicating dependence on H_2_O_2_) and even causes platelet aggregation [56]. Antibacterial testing of LAAOs that were purified from *Daboia russellii siamensis* venom revealed the strongest activity against *S. aureus* (MIC = 9.0 μg/mL), while higher MICs were needed for *P. aeruginosa* (144.0 μg/mL) and *E.coli* (288.0 μg/mL). Against eight clinical MRSA isolates, MICs ranged from 4.5 to 36.0 μg/mL and MBCs from 9.0 to 72.0 μg/mL, confirming potent activity, particularly against Gram-positive strains [57]. The LAAO of *Cerastes cerastes* exhibited antibacterial activity against *S. aureus*, MRSA, and *P. aeruginosa*, with MIC values of 10, 10, and 20 μg/mL, respectively. No activity was detected against *E. coli* or yeast [58]. LAAO isolated from *Crotalus durissus cumanensis* venom produced H_2_O_2_ and showed dose-dependent antibacterial activity, with MIC/MBC values of 8/16 μg/mL for *S. aureus* and 16/32 μg/mL for *A. baumannii*. No activity was observed against *E. coli* [59]. Interestingly, *Naja naja oxiana* LAAO in the process manifests antibacterial activity against both *B. subtilis* (Gram-positive) and *E. coli* (Gram-negative) [60]. LAAOs from *Bothrops moojeni* and *Bothrops jararacussu* have exhibited MIC values as low as 0.12 µg/mL against *S. aureus* and *E. coli*, highlighting their potential as powerful antimicrobial agents [61].

### 2.7. Antimicrobial Peptides (AMPs)

Snake venoms are complex biological mixtures containing an odd assortment of bioactive molecules, some of which exhibit vigorous antibacterial activity and hold immense potential as future alternatives to conventional antibiotics in the age of escalating antimicrobial resistance [62]. Among such compounds, AMPs such as cathelicidins have been of considerable interest due to their broad-spectrum activity against Gram-positive and Gram-negative bacteria [63]. Similarly, studies on cathelicidin NA-CATH, for example, demonstrated its antibacterial potential against *S. aureus* [64], and although this compound did not show hemolytic activity in horse erythrocytes, it displayed antibacterial efficacy with an MIC of 2.9 µg/mL [64]. Further studies by Blower et al. (2015) showed its potential against *Burkholderia thailandensis*, but it failed to eradicate preformed biofilms [65]. Other snake venom-derived peptides, such as cathelicidin-BF, OH-CATH, and Crotalicidin (Ctn), have also demonstrated strong antibacterial activities against multidrug-resistant bacteria. Cathelicidin-BF, in particular, exhibited potent activity against a variety of pathogens, including Gram-negative bacteria [66], and the presence of salts like NaCl in the solution enhanced its antimicrobial efficacy [67]. Similarly, OH-CATH, derived from Asian snakes, has shown strong antimicrobial properties against both standard and multidrug-resistant clinical isolates with MICs ranging from 1.56 to 12.5 μg/mL, and against clinical isolates of *E. coli*, *P. aeruginosa*, and MRSA [68], improving survival in a bacteremia animal model induced by drug-resistant *E. coli*.

The peptides Pt_CRAMP1, Ctn, and BatxC exhibited a better inhibition of Gram-negative pathogens compared to other peptides [69]. Specifically, Ctn and its fragment Ctn [15,16,17,18,19,20,21,22,23,24,25,26,27,28,29,30,31,32,33,34] were found to be bactericidal against Gram-negative bacteria like *E. coli* and *P. aeruginosa*, showing a significant reduction in bacterial cell populations within a relatively short timeframe. This suggests a strong antimicrobial activity against these specific bacterial species [69]. In contrast, Ctn and BatxC demonstrated no hemolytic effects while effectively inhibiting clinical isolates of *K. pneumoniae* [70].

In addition to their antibacterial properties, these peptides, especially CATHPb1, have shown considerable anti-biofilm activity. CATHPb1 was effective in preventing biofilm formation and eradicating preformed biofilms in *E. coli*, *P. aeruginosa*, *K. oxytoca*, and *S. aureus* [71]. Its broad-spectrum activity, low cytotoxicity, and ability to combat biofilm-associated infections make it a promising candidate for therapeutic applications in the fight against multidrug-resistant pathogens. Interestingly, an antibacterial peptide purified from *Naja naja* venom, with a molecular weight of 2491 Da and N-terminal sequence DEQSTHGAYVWKL, exhibited strong activity against both Gram-negative and Gram-positive bacteria without hemolytic activity and thus has potential for therapeutic application [72]. Synthetic analogs have also been engineered in recent studies, like the cationic peptide SP1V3_1, which display broad-spectrum activity with immune-modulatory and anti-biofilm activity, in addition to activity against drug-resistant bacteria like MRSA and *K. pneumoniae* [73]. Certain other venom-derived peptides, like pC-CoaTxII from *Crotalus oreganus abyssus,* display potential against multidrug-resistant bacteria by creating pores in microbial membranes [74].

### 2.8. Other Proteins

**Figure 1:**
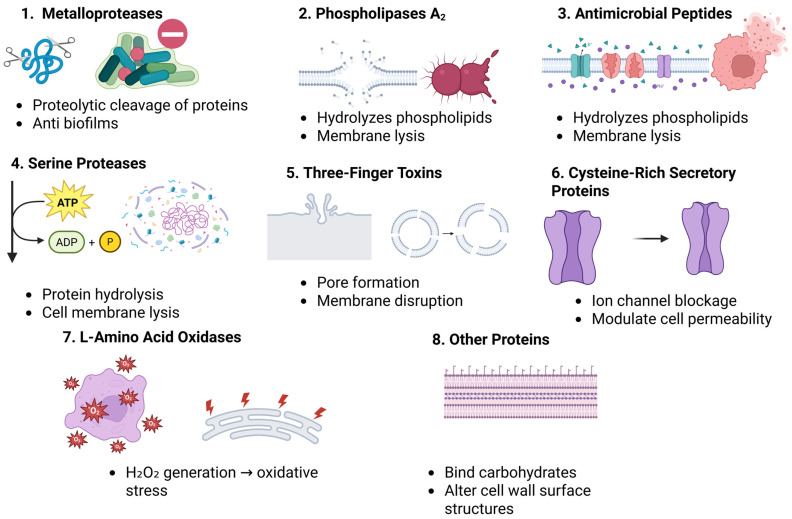
This figure summarizes the structures and antibacterial activities of eight major snake venom toxins: MPs, SPs, PLA_2_s, 3FTxs, CRISPs, LAAOs, lectins, and peptides. Each panel illustrates the molecular architecture of the toxin class and outlines its proposed antibacterial mechanisms, such as membrane disruption, enzymatic degradation, inhibition of bacterial growth, or biofilm suppression.

In addition to the aforementioned protein families, several other snake venom components display antibacterial activity. Notable examples include PaTx-II, member of the PLA_2_ from *Pseudechis australis*, which inhibits *S. aureus* and *Proteus vulgaris* with an MIC of 25 µM [75] and cardiotoxin 3 from *Naja atra*, which exhibits strong activity against *S. aureus* [76]. Cardiotoxin 1 (CTX-1) from *Naja atra* (Chinese cobra) inspired the design of short antimicrobial peptides with reduced toxicity. Among them, NCP-3 and its variants showed strong activity against bacteria, fungi, mycobacteria, and viruses, with low cytotoxicity and stability in high-salt conditions. These findings suggest that toxin-derived peptides are promising candidates for novel antimicrobial agents [77]. *Naja naja atra* CTX3 and *Naja nigricollis* toxin γ kill bacteria by disrupting membranes. This study found their membrane-damaging activity is linked to their ability to fuse with bacterial membrane mimics. CTX3 showed stronger activity on cardiolipin-rich membranes (like in *S. aureus*), while both toxins altered membrane structure and fluidity [78]. Together, these findings underscore the immense potential of snake venom-derived proteins as a rich source of novel antimicrobial agents. Further research into their mechanisms of action, toxicity, and therapeutic applications could pave the way for innovative treatments against multidrug-resistant bacterial infections [13]. Table 1 summarizes essential information regarding the venom components, their antibacterial targets, mechanisms, examples, and MICs against various bacteria. Figure 1 shows the antibacterial mechanisms of snake venom toxins.

## 3. Antimicrobial Mechanisms of Snake Venom Components

Snake venom components exert their antibacterial activities by taking on different mechanisms, ranging from direct membrane disruption to immunomodulatory functions. These functions not only affect planktonic bacterial cells but also target biofilms, which are notoriously resistant to antibiotics [79]. Elucidating these antimicrobial mechanisms is paramount in the development of snake venom-based compounds as therapeutic medicines for the treatment of multidrug-resistant bacteria [15].

### 3.1. Membrane Disruption and Lysis

Snake venom gains its antimicrobial effect primarily through membrane disruption, by proteins such as PLA_2_s, 3FTxs, and AMPs. The venom proteins are cationic and amphiphilic and hence can be electrostatically attracted to the negatively charged bacterial membranes, particularly those that are rich in lipopolysaccharides (Gram-negative) or teichoic acids (Gram-positive) [80]. Upon binding, their amphiphilic character allows the hydrophobic region to penetrate into the lipid bilayer and hydrophilic regions to engage with the aqueous environment [81]. PLA_2_s hydrolyze membrane phospholipids to yield lysophospholipids and free fatty acids, leading to increased membrane permeability and thinning [82]. 3FTxs can insert into membranes through their loop domains and exert curvature, pore creation, or lipid phase segregation [41]. AMPs operate with various models of disruption: barrel-stave forms transmembrane pores, toroidal pore bends lipids to form ring-shaped pores, and carpet models lead to the complete breakdown of membranes [83]. Such disruptions lead to leakage of ions, membrane depolarization, the loss of cytoplasmic material, and the disruption of bioenergetic gradients [84]. Lastly, the bacterial cell is ruptured by osmotic disruption and membrane failure [85]. Further, other venom components such as LAAOs also contribute by generating hydrogen peroxide, again triggering oxidative stress [52]. Such mechanisms tend to be synergistic, so the venom of snakes is a very effective natural antimicrobial compound via direct and multi-faceted targeting of bacterial membranes [52].

### 3.2. Enzymatic Breakdown of Bacterial Components

Snake venoms possess potent antibacterial action via enzymatic mechanisms that work by damaging crucial bacterial structures, primarily via membrane disruption and oxidative damage [86]. Of these, PLA_2_s hydrolyze the sn-2 acyl bond of phospholipids in bacterial membranes to produce lysophospholipids and free fatty acids, which compromise membrane integrity and fluidity, leading to increased permeability, ion leakage, and ultimately bacterial lysis [82]. This perturbation also perturbs membrane-bound proteins that are essential for nutrient uptake and energy generation. At the same time, LAAOs catalyze the oxidative deamination of L-amino acids to α-keto acids with the release of ammonia and the formation of hydrogen peroxide (H_2_O_2_) as a by-product. H_2_O_2_ causes oxidative stress in bacterial cells by damaging nucleic acids, proteins, and membrane lipids, further enhancing membrane instability and inhibition of metabolic processes [87]. In addition to these, MPs have the capability to degrade bacterial surface proteins and extracellular matrices, impacting structural integrity and potentially disrupting biofilm formation [12]. The combined action of these enzymes not only lysates the bacterial cells but also can interfere with quorum sensing and microbial colonization [88]. SPs aid in bacterial breakdown by degrading structural proteins, disrupting biofilm structure, and preventing bacterial adhesion to host cell [89]. In concert, the process entails initial binding to membranes and lipid hydrolysis by PLA_2_, subsequent oxidative injury from H_2_O_2_ produced by LAAO, and structural disruption through MPs, leading to the death of bacterial cells [90].

### 3.3. Induction of Oxidative Stress

Snake venoms and its compounds confer high antimicrobial activity by destroying bacterial membranes, inducing oxidative stress, and compromising bacterial cell wall integrity. Oxidative stress triggered by the venom involves the generation of reactive oxygen species (ROS), leading to tissue damage and systemic effects like acute kidney injury, hemorrhage, and inflammation [91]. LAAO catalyzes the oxidation of L-amino acids, which produces H_2_O_2_. The accumulation of H_2_O_2_ inside the bacterial cell induces oxidative stress, interacting with cellular components like proteins, lipids, and DNA [92]. This leads to significant damage within the bacterial cell, including the weakening of the bacterial cell wall, which makes it more vulnerable to disruption. Simultaneously, PLA_2_ acts on the bacterial cell membrane, hydrolyzing phospholipids, which are essential components of the membrane’s structure. The enzymatic activity of PLA_2_ destabilizes the membrane, leading to the leakage of cellular contents and eventually causing cell lysis, which results in bacterial death [93]. This dual mechanism—where oxidative stress induced by LAAO weakens the cell structure and PLA_2_ disrupts the membrane integrity—synergistically leads to effective bacterial killing [90]. Furthermore, many snake venoms also contain AMPs, which possess direct antibacterial activity by targeting bacterial membranes and further enhancing the venom’s antimicrobial efficacy [94]. This multi-pronged approach, combining ROS generation, membrane disruption, and the action of antimicrobial peptides, ensures the venom’s potency in combating bacterial infections. Such oxidative stress pathways are particularly effective against antibiotic-resistant bacteria since they disrupt the bacterial redox homeostasis and render the bacteria more susceptible to host immune attack [95]. Oxidative stress can also enhance the effectiveness of conventional antibiotics by weakening bacterial defenses [96].

### 3.4. Inhibition of Biofilm Formation

Snake venoms have a remarkable capacity to block the development of biofilms, the process whereby bacteria attach to surfaces and create resistant, ordered communities [12]. Biofilms are notoriously intractable to treatment and play a role in chronic infections. The antimicrobial activity of the venom, notably its capacity to damage bacterial cells and block the development of biofilms, arises from multiple mechanisms, each of which contributes to the inhibition of biofilm formation and to the breakdown of established biofilms [97]. Snake venom inhibits biofilm formation through several mechanisms that target the integrity and behavior of bacterial cells. One of the primary ways this occurs is through cell membrane disruption, where enzymes like PLA_2_ hydrolyze the phospholipids in bacterial membranes [30]. This destabilizes the membrane, increasing its permeability and preventing bacterial adhesion to surfaces, a critical first step in biofilm formation. Additionally, interfering with cell division plays a key role, as certain venom peptides, such as pseudonajide, break down bacterial cell walls and membranes, causing morphological defects and preventing proper cell division [98]. Without successful division, the bacterial population cannot grow or form a stable biofilm. Interfering with extracellular matrix (ECM) formation is another significant effect of snake venom, particularly through enzymes like LAAO, which produce ROS, such as hydrogen peroxide. These ROS cause oxidative stress within bacterial cells, leading to alterations in the cell surface and hindering the formation of the ECM, which is essential for the structure and protection of mature biofilms [99]. By preventing ECM development, venom components make bacteria more susceptible to antimicrobial agents. Furthermore, inhibition of bacterial adhesion is achieved by venom components that interfere with bacterial adhesins or surface proteins, which are responsible for initial surface attachment. Without this adhesion, biofilm formation is effectively blocked [100]. Venom can also disrupt cell-to-cell signaling within biofilms by interfering with quorum sensing, a process through which bacteria communicate to regulate the formation of biofilms and the expression of virulence factors. By preventing this coordinated behavior, venom components stop bacteria from forming organized biofilm structures [39]. Finally, degradation of the biofilm matrix occurs via venom proteases, which break down extracellular polymers such as proteins and polysaccharides that form the structural backbone of biofilms [12]. This degradation weakens the biofilm, allowing antimicrobials or immune responses to penetrate more effectively and eliminate the bacteria within. Together, these mechanisms make snake venom a potent inhibitor of biofilm formation and bacterial survival. These venom components provide a multi-modal mechanism of preventing biofilm development through action at multiple stages of the biofilm life cycle, ranging from bacterial adhesion to matrix formation and cell signaling [101]. Antimicrobial and anti-biofilm functions of snake venoms are therefore of great interest for identifying new therapeutic paradigms against biofilm-based infections, typically recalcitrant to antibiotics.

### 3.5. Immunomodulatory Effects Against Bacterial Infections

Besides direct antimicrobial actions, snake venom components also modulate the host immune response to infection by bacteria and regulate complex immune signaling pathways [102]. For example, the enzymes MPs, SPs, and PLA_2_ found in snake venom may be able to trigger pro-inflammatory responses, activate the complement system, and lead to the release of damage-associated molecular patterns (DAMPs) [103]. These components facilitate the recruitment and activation of key innate immune cells, including neutrophils, macrophages, and dendritic cells, which are essential for initiating early responses against invading pathogens [12]. Mechanistically, these venom-derived molecules can engage pattern recognition receptors (PRRs), particularly the toll-like receptors (TLRs). For instance, activation of the TLR2/MyD88 signaling axis by PLA_2_s enhances the expression of pro-inflammatory cytokines such as TNF-α, IL-1β, and IL-6. This leads to amplified phagocytic activity and improved bacterial clearance by macrophages [104]. This adjuvant-like effect highlights the potential of venom components in boosting innate immunity during infections or even in vaccine development [105]. Proteins such as snake venom cathelicidins, which have shown both antibacterial and anti-biofilm activities, are of particular interest because they can modulate the innate immune response, with potential use in both antimicrobial therapy and immune modulation [106]. Furthermore, research on the immunomodulatory and antimicrobial activities of venom components elucidates their mechanisms of action. Venom proteins also are capable of activating NF-κB and TGF-β1/Smad signaling pathways, which play significant roles in inflammation and wound healing. Such dual role of snake venoms as strong modulators of immunity and antimicrobial compounds creates opportunities for their use in infection treatment and immune disorders, ranging from cancer immunotherapy to the identification of novel antibacterial agents against drug-resistant bacteria [107]. In another study, some venom components, particularly group IIA PLA_2_s such as MT-III from *Bothrops asper* and BthTX-I from *Bothrops jararacussu*, play a dual role in modulating inflammation. These enzymes have been reported to activate the NLRP3 inflammasome complex in macrophages, a critical platform for the maturation and release of interleukin-1β (IL-1β), a key cytokine in antimicrobial defense [108]. In parallel, these same components induce the expression of cyclooxygenase-2 (COX-2) and promote the synthesis of bioactive lipid mediators, including prostaglandin E2 (PGE2) and 15-deoxy-Δ12,14-prostaglandin J2 (15-d-PGJ2) [109]. These lipid mediators are involved in both the amplification of inflammation and its subsequent resolution, suggesting a tightly regulated, context-dependent modulation of immune responses by venom toxins [110]. Such dual-function immunomodulation—both triggering acute inflammation and facilitating its resolution—indicates that snake venom molecules might act as fine-tuners of host defense. This property can be exploited therapeutically to restore immune balance in chronic infections or inflammatory diseases where either excessive or insufficient immune responses are detrimental.

**Figure 2:**
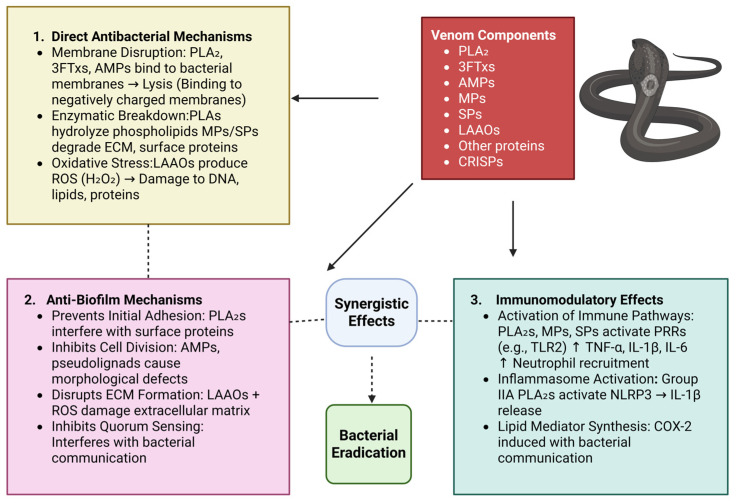
The flowchart illustrates how snake venom components—PLA_2_s, LAAOs, 3FTxs, AMPs, MPs, and SPs—exert antimicrobial effects through three main pathways: (1) Direct antibacterial actions via membrane disruption, enzymatic degradation, and oxidative stress; (2) Anti-biofilm mechanisms that prevent adhesion, disrupt quorum sensing, and degrade the matrix; (3) Immunomodulatory effects, including immune activation, inflammasome triggering, and lipid mediator synthesis. Synergistic interactions contribute to effective bacterial eradication.

Overall, compounds from snake venom exhibit multifaceted antimicrobial activities, including membrane disruption, enzymatic hydrolysis, induction of oxidative stress, biofilm inhibition, and immune modulation. Compounds from venom are promising leads for antibiotic-resistant bacterial infection because of these multifaceted mechanisms. Investigation of their mechanism of action, safety, and clinical usefulness would further translate into novel antimicrobial therapeutics. Figure 2 illustrates how snake venom components—PLA_2_s, LAAOs, 3FTxs, AMPs, MPs, and SPs—exert antimicrobial effects through three main pathways.

## 4. Extracellular Vesicles: Types, Composition, and Functional Roles

Extracellular vesicles (EVs) are lipid bilayer-vesicles of nano-sized origin secreted from various cell types, facilitating intercellular communication by transferring bioactive molecules such as proteins, lipids, and nucleic acids [111]. Traditionally, EVs are classified into three major groups: exosomes (40–100 nm), microvesicles (100–1000 nm), and apoptotic bodies (500–5000 nm) with varying distinctions in biogenesis and function [112]. There are recently discovered subtypes in the recent findings, which include autophagic EVs, matrix vesicles, and stressed EVs involved in diverse physiological and pathological phenomena [113]. The molecular composition of EVs is a reflection of their cellular origin, with key biomolecules influencing immune regulation, angiogenesis, and cancer growth. Exosomes, in particular, are tetraspanin-positive (CD9, CD63, and CD81), integrin-positive, full of regulatory RNAs, and play a significant role in disease pathophysiology, e.g., cancer metastasis and drug resistance [114,115]. Their lipid contents, such as sphingomyelin, phosphatidylserine, and ceramides, also contribute to membrane stability and signaling. EVs have been identified as candidate biomarkers and therapeutic tools owing to their stability in biofluids and ability to ferry molecular cargo across cells [116]. High-throughput omics technological advances have put exosomes on the front lines as putative biomarkers for disease diagnosis and progression [117]. Exosomes’ resistance to instability in biofluids and their ability to penetrate biological barriers make them promising agents for non-invasive liquid cancer, neurodegenerative disease, and infectious disease biopsies [118]. Furthermore, exosomes are currently under investigation as therapeutic delivery vehicles due to their ability to be biocompatible, have intrinsic targeting properties, and deliver drugs, siRNA, mRNA, and even CRISPR-Cas9 for precision medicine [119]. Isolation involves techniques such as ultracentrifugation, size-exclusion chromatography, and microfluidic-based separation, and characterization via electron microscopy, nanoparticle tracking analysis, and flow cytometry [120]. EVs induce immunosuppression, metastasis, and drug resistance in cancer and represent potential targets for diagnosis and therapy. With research becoming increasingly innovative, EV-based strategies have immense potential for precision medicine and targeted drug delivery [121]. Despite being promising, exosome-based therapies are still in the process of being brought to the clinic. Among the largest hurdles are standardization of methods for isolation, overcoming heterogeneity, scaling large-volume production, and regulatory affairs [122]. Advances nonetheless in EV engineering and characterization still drive exosome-based technologies to future applications of precision medicine with new opportunities in diagnostics and targeted therapeutics.

### 4.1. The Role of Extracellular Vesicles in the Amplification of Snake Venom-Derived Antibacterial Therapies

Snake venom-derived extracellular vesicles (SVEVs) represent a new and emerging avenue for the development of next-generation antibacterial therapies, particularly for addressing the vital challenge of MDR bacterial infections [123]. Although first reported in snake venoms in 1973, more recent research has also identified and characterized similar vesicles in bee, wasp, spider, and tick venom [124]. EV separation and enrichment from venom must be highly specialized and utilize methodologies such as differential and ultracentrifugation, size exclusion chromatography (SEC), extracellular vesicle total recovery and purification (EVTRAP), polymer-based precipitation, and antibody-based capture methodologies [125].

SVEVs secreted by snake venom glands possess an extensive range of bioactive compounds including AMPs, enzymes such as PLA_2_s and LAAOs, and bioactive toxins. As natural nano-sized lipid bilayer-enclosed particles, SVEVs protect their therapeutic load from enzymatic degradation and immune clearance, thus allowing effective and targeted delivery to infection sites [13,126]. Aside from enhancing the stability and bioavailability of unstable venom-derived molecules, this vesicular entrapment enhances their antimicrobial potency by promoting synergistic action and sustained release. Most importantly, the various mechanisms of action inherent to SVEVs, ranging from membrane damage and oxidative stress induction to enzyme-catalyzed bacterial component degradation, significantly reduce the possibility of resistance development, a significant advantage over traditional antibiotics [123]. SVEVs’ ability to penetrate across biological barriers and biofilms promises potential for topical wound healing, the systemic treatment of sepsis, and adjuvant therapies to increase the efficacy of traditional antibiotics [127]. Some of the remarkable antimicrobial substances transported by SVEVs are cathelicidins, defensins, PLA_2_, and LAAOs, which have proven to exhibit stronger antibacterial activities when formulated as vesicles since they gain improved targeting and protection [128]. However, the translation of SVEVs to the bedside depends on overcoming several challenges such as developing standardized and scalable methods for their purification and isolation, performing extensive proteomic and lipidomic characterization to determine their functional constituents, attaining biosafety by removal of cytotoxicity hazard, and assuring their therapeutic efficacy and safety by rigorous in vivo assessment and clinical trials [129]. In spite of these challenges, SVEVs present an innovative and biocompatible scaffold with inherent antimicrobial properties, making them a strong new generation of biogenic nanocarriers for combating antibiotic resistance [130].

### 4.2. Extracellular Vesicles as Delivery Vessels for Snake Venom-Derived Antibacterial Compounds

SVEVs are emerging as next-generation natural nanocarriers for antibacterial drug delivery because of their unique biological origin and inherent cargo-carrying ability [123]. Membrane-bound nanosized vesicles, released from venom gland epithelial cells, naturally encapsulate a variety of bioactive molecules like enzymes, antimicrobial peptides, and lipids, which imbue them with intrinsic therapeutic potential [131]. The ability of SVEVs to be engineered for encapsulating and targeting the delivery of antibacterial drugs—such as cathelicidins and defensins, which are abundant in snake venom—adds to their appeal as delivery vehicles [70]. Their lipid bilayer structure is protective to the encapsulated therapeutic drugs from enzymatic degradation and enhances their stability, bioavailability, and delivery to infected tissues [132]. Furthermore, SVEVs have been functionalized with targeting ligands or antibodies for increasing specificity toward bacterial pathogens or infected host cells in order to reduce systemic toxicity and off-target effects [133]. Preliminary studies, including those involving *Bothrops jararaca* derived SVEVs, have demonstrated the presence of antimicrobial proteins like 5′-nucleotidases and L-amino acid oxidases in support of the possibility that SVEVs can generate synergistic bactericidal activities as carriers [134]. Additionally, their generally low immunogenicity and biocompatibility also confer a safety advantage over most synthetic drug delivery systems [135]. Exosome-based delivery has the capability of making the venom-derived peptides more specific, attacking bacterial infections and not host tissues to a great extent. Exosomes have intrinsic targeting capabilities that are additionally tunable through surface engineering [136]. Functionalization by the attachment of bacteria-targeting ligands, i.e., antibodies or peptides, has the potential to enhance the selectivity of venom-derived antimicrobial drugs towards pathogenic bacteria [137]. The integration of venom research and extracellular vesicle technology holds promising directions for the creation of SVEV-based antibacterial therapies, particularly to counteract antibiotic resistance and enhance the specificity of antimicrobial therapies. Table 2 explains the biological characteristics, bioactive components, mechanisms of action, advantages, and current challenges associated with the use of SVEVs for combating bacterial infections, particularly MDR strains.

## 5. Possible Strategies to Improve Antibacterial Activity of Snake Venom

PEGylation has been an interesting strategy to enhance the therapeutic potential of snake venom toxins and AMPs through improved pharmacokinetics, reduced immunogenicity, and endowing molecular stability [138]. Studies of snake venom SP, such as SPCdc and rCollinein-1, demonstrated that PEGylation could be utilized for maintaining or augmenting enzymatic activity and substrate binding, reducing immunogenicity, and retaining structural stability [138,139]. In the case of AMPs, PEGylation typically reduces antibacterial activity by preventing membrane interaction but reduces hemolytic toxicity by far, hence enhancing therapeutic indexes [140]. Interestingly, PEGylated peptides like Bac7(1-35) do not lose bacterial cell penetration and use the same transporters as their native versions [141]. Further, PEGylation reduces renal clearance and in vivo bioavailability, making the technique a worthy candidate for new antimicrobial drug and biopharmaceutical development from snake venom toxins [142].

Liposomes have proved extremely promising as snake venom carriers for protective immunization, with safer and more effective alternatives to the traditional antivenom production method [143]. The vesicles based on phospholipids can deliver a broad spectrum of venom components, including venomous enzymes such as phospholipases, while minimizing toxicity and enhancing immunogenicity [144]. Various formulations have been tested, including those using dimethyl dioctadecyl lammonium bromide and cholesterol, which had high encapsulation efficiency and stability [145]. Approaches like venom modification and membrane stabilization—e.g., with osmium tetroxide or native phospholipids—have addressed problems of venom-induced liposomal lysis [146]. These approaches have elicited highly effective, sustained immune responses in animal models including sheep, mice, and rabbits following intravenous, subcutaneous, or even oral administration [147]. Notably, immunostimulant-containing preparations further enhanced antibody levels and protective capacity [148]. In addition, liposomes have been used to administer individual PLA_2_ enzymes, for targeted antimicrobial therapy, suggesting dual utility for combating drug-resistant infections [149]. Overall, liposomal encapsulation maximizes the safety and efficacy of venom immunization but also offers avenues for new therapeutic applications.

The integration of snake venom components into hydrogel systems represents a novel and promising approach for creating bioactive materials with potent antibacterial properties and applications in wound healing, infection control, and hemostasis [150]. These hybrid biomaterials harness the natural antimicrobial potency of snake venom enzymes and peptides, as well as the controlled delivery and biocompatibility of hydrogels [151]. Hydrogels are hydrophilic three-dimensional polymeric networks that are able to absorb large quantities of water without compromising structural integrity. Their biocompatibility, ability to have tunable mechanical properties, and responsiveness to environmental cues make them suited for biomedical use [152]. In biomedical applications, venom-containing hydrogels are advanced wound dressings that inhibit bacterial infection, biofilm formation, and enhance tissue repair by facilitating increased angiogenesis and cell migration [153]. Additionally, some venom constituents also exhibit immunomodulatory and regenerative activities, vouchsafing their application for curing chronic wounds and tissue engineering. The key advantages of snake venom-loaded hydrogels include sustained release of the antibacterial agents, reduced systemic toxicity through local delivery, multifunctionality with multimodal combination of antimicrobial, hemostatic and regenerative activities, and versatility for the treatment of wounds of varying severity and type [154,155].

Microneedle patches offer a minimally invasive yet highly efficient platform for transdermal drug delivery, able to penetrate through skin and biofilm barriers to deliver therapeutic agents to infected or injured tissue [156]. Recent developments in microneedle technology borrow from recent breakthroughs in natural systems such as the fangs of snakes that utilize open-groove architectures to achieve rapid liquid delivery through capillary action [157]. They have been utilized to deliver vaccines, antibiotics, and heat-killed bacteria for vaccination and infection prevention [158]. Integration of venom-derived bioactive molecules from snake venom into microneedle systems is a novel and promising avenue. Venom-derived compounds can be loaded into dissolving or responsive microneedles to provide localized, controlled delivery with low systemic toxicity and high therapeutic efficacy [159]. When combined with enzymolysis or photothermal agents, microneedles loaded with snake venom components could offer triple-combination therapy for stubborn biofilm-associated infections, similar to the approach demonstrated by Yu et al. (2022) but with enhanced potency due to the multifunctionality of venom constituents [160]. A study has shown that by incorporating microneedles and MgB_2_ microparticles (MgB_2_ MN), it is possible to kill over 5 log of bacteria, neutralize acidity, and reduce inflammation by binding to bacterial LPS [161]. This approach opens the possibility of exploring the use of snake venom peptides, creating a powerful, multifunctional strategy for treating bacterial infections.

Biopolymer films have proven to be multifunctional platforms for the building of new antimicrobial solutions, especially by incorporating bioactive ingredients such as living microbes or venom-derived components [161]. Better still, active biopolymer films like those constituting collagen, sodium alginate, and sorbitol (CA-S) have been shown to be able to store and release *Bdellovibrio bacteriovorus*, a predator bacterium with specificity to pathogens, to effectively lyse Gram-negative pathogens such as *E. coli* [162], positioning such films as potential solutions to microbial control in food packaging and clinical settings. Parallel research has taken advantage of the particular biochemical characteristics of snake venom enzymes, such as PLA_2_, entrapped in Langmuir–Blodgett films along with carbon nanotubes to permit the stable preservation of enzymic architecture and catalytic antimicrobial properties [163]. In addition, recombinant protein engineering advancements have enabled the development of biopolymer films with functionalization of AMPs, resulting in broad-spectrum activity against Gram-negative and Gram-positive bacteria [164]. These disparate platforms have intriguing uses: AMP-functionalized protein polymers are under development for infection-resistant wound dressings; venom PLA_2_ and proteomes show potential in the prevention of biofilm; and venom-derived AMP-based controlled-release systems are being explored to modulate infection in medical devices [165]. Collectively, these studies highlight the vast therapeutic and industrial value of biopolymer films with living organisms or venom-derived biomolecules, offering a novel, biodegradable, and efficient tool to combat microbial contamination and antibiotic-resistant infections in biomedical and food uses.

**Figure 3:**
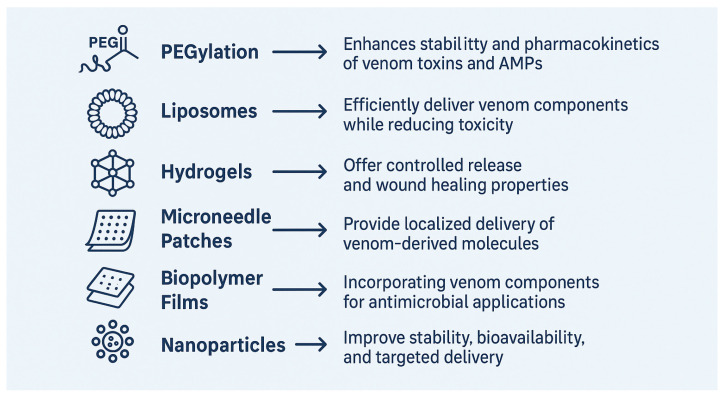
Possible strategies to improve the antibacterial activity of snake venom. This illustration highlights modern drug delivery approaches designed to improve the therapeutic potential of snake venom-derived antimicrobial agents. PEGylation enhances the pharmacokinetics and stability of venom toxins and antimicrobial peptides (AMPs). Liposomes facilitate efficient delivery while minimizing toxicity. Hydrogels support controlled release and possess wound-healing capabilities. Microneedle patches enable localized administration of venom compounds. Biopolymer films offer a platform for integrating venom components in antimicrobial applications. Nanoparticles further enhance stability, bioavailability, and targeted delivery, collectively contributing to more effective antibacterial therapies.

Trapping snake venom in nanoparticles, more precisely in polymeric carriers such as chitosan or PLGA-based systems, represents a new strategy to enhance the therapeutic potential of venom components, especially for antibacterial application [166]. The approach has the benefit of enhancing the stability, bioavailability, and targeted delivery of venom biomolecules while minimizing systemic toxicity, and as a result, constitutes an interesting avenue for the future development of new antimicrobial therapies [167]. Nanoparticles function as efficient carriers by protecting labile venom compounds from enzymatic degradation, fostering controlled release, and enabling selective accumulation in infection sites or target tissues [168]. Chitosan nanoparticles, biocompatible and mucoadhesive, have been used effectively to encapsulate snake venoms, and these systems significantly amplify the antibacterial potency of the venoms [169]. A case in point is *Bothrops jararacussu* venom, whose encapsulation in nanoparticles leads to enhanced antibacterial activity, particularly against Gram-positive bacterial strains such as *S. aureus* [165]. Similarly, chitosan nanoparticles, with their biocompatibility and intrinsic antimicrobial activity, have been reported to encapsulate *Echis carinatus*, and *Crotalus durissus cascavella* venom proteins, enhancing antibacterial efficiency and reducing inflammation [165]. Altogether, these chemical and nanotechnological modifications are transforming snake venom-derived peptides as useful therapeutic agents against multidrug-resistant infections, offering a new and effective means to combat the growing menace of antimicrobial resistance. Nanoparticle encapsulation not only preserves these mechanisms but also facilitates their localized action, thereby reducing the likelihood of systemic side effects. The benefits of this encapsulation method are not only for antimicrobial therapy; the enhanced biodistribution and residence time of venom molecules also promise the potential for more effective treatments of infection, even defying antibiotic resistance [170]. Table 3 shows the advantages and applications of various delivery systems for snake venom components. Figure 3 shows the possible strategies to improve the antibacterial activity of snake venom.

## 6. Enhancing Antibacterial Activity Through Peptide Modification

One of the strongest methods to enhance the antibacterial activity of peptide-derived compounds from snake venom is rational design and structure modification, which can be utilized to optimize their pharmacokinetics and pharmacodynamics while either retaining or improving antimicrobial activity [171]. Amino acid substitution is generally utilized to modulate important features such as hydrophobicity, amphipathicity, and net positive charge, all of which can have a profound effect on the interaction of the peptide with bacterial membranes [172]. Raising the number of hydrophobic or positively charged residues can lead to increased electrostatic interactions and improved membrane disruption, yielding elevated bactericidal activity [67]. Insertion or deletion mutations can also be utilized to regulate the secondary structure, for example, α-helices or β-sheets or even overall flexibility of the peptide, which can set the stage for whether it penetrates and destabilizes microbial membranes [106]. A substitution of cysteine with alanine suppresses disulfide bond formation, preserving antimicrobial activity, while enhancing peptide stability. For instance, for the peptides crotamine and bothropstins [173], replacing cysteine with alanine retains their antimicrobial efficacy while improving their structural robustness, making them more suitable for therapeutic applications. Similarly, the incorporation of tryptophan residues, characterized by membrane-disruptive effects, into the peptides melittin and vipericidins enhances penetration across the membrane as well as cell destabilization by bacteria [174]. The insertion of positively charged residues increases electrostatic interactions with negatively charged bacterial membranes, improving penetration and antimicrobial efficacy, especially against Gram-positive and drug-resistant bacteria [175]. Shortened cathelicidins such as LZ1 and ZY13 from cathelicidin-BF retained strong antimicrobial activity with improved pharmacokinetic profiles. Directed evolution approaches impose random mutations to generate AMP variants with increased binding affinity, enhanced broad-spectrum activity, and increased proteolytic resistance. Synthetic analogs of venom-derived AMPs are also engineered to enhance selectivity, efficacy, and in vivo stability, thereby improving their performance under physiological conditions. All combined, these targeted alterations represent an exciting frontier for the development of very potent and long-acting peptide-based therapeutics from snake venom, particularly in the fight against multidrug-resistant bacterial infections.

Reducing the toxicity of snake venom for biomedical applications is a complex process that enhances therapeutic use without enhancing side effects [176]. The isolation and modification of venom components to identify non-toxic or low-toxicity molecules with pharmaceutical value is one of the main strategies [8]. This includes the bradykinin-potentiating peptides (BPPs), which were employed to discover antihypertensive drugs such as Captopril, and fibrinogenolytic and fibrinolytic enzymes with the possibility of being utilized in the treatment of cardiovascular disorders and thrombosis [177]. Toxic components can be genetically or chemically modified to maintain therapeutic efficacy with reduced systemic toxicity. Other natural inhibitors from animal sera or plant material have shown neutralizing action against venom enzymes like PLA_2_ and metal chelators like EDTA can inhibit metalloproteinases by dislodging necessary metal ions [178]. The use of monoclonal antibodies (mAbs) targeted to specific toxins in the venom is another hopeful area of research that provides highly specific, low-immunogenicity alternatives to traditional antivenoms [179]. Recent advancement in antibody engineering has enhanced the safety and efficacy of mAbs in targeted neutralization without cross-reactivity [180]. Nanoparticle-based methods also improve delivery of the venom components by sequestering the toxins for target and controlled release, reducing off-target effects and improving therapeutic index [181]. Theranostic use of these nanoparticles is also feasible by functionalizing them for therapy in addition to diagnostic tracking. Other strategies include combination therapies where antivenoms are paired with mAbs, nanoparticles, or natural inhibitors for synergistic effect, and immunization and vaccine development from inactivated venom components to elicit protective immunity [142]. Furthermore, certain peptides derived from snake venom have been discovered to exhibit antimicrobial activity, which has potential as novel drugs against multidrug-resistant infections. All these new methods are redefining drug discovery from venoms to become relevant to a wide range of diseases including hypertension, bleeding disorders, infection, and cancer, with much less risk of associated toxicity. Table 4 summarizes key approaches to enhance the antibacterial efficacy of venom-derived peptides, including amino acid substitution, structural modifications, and synthetic analog design. It also highlights strategies to reduce toxicity, such as component isolation, genetic and chemical modifications, the use of natural inhibitors, monoclonal antibodies, nanoparticle delivery systems, and immunization approaches, aiming to improve therapeutic potential and clinical safety.

## 7. Conclusions and Future Direction

On the whole, this review provides critical insights on the antimicrobial efficacy of snake venom proteins which show diverse modes of action involving membrane disruption, enzymatic destruction, oxidative stress induction, and inhibition of biofilm formation. These mechanisms underscore the promising future of venom proteins as novel therapeutic agents, particularly in combating multidrug-resistant infections. The review also comes to highlight the new concept of SVEVs as high-performance carriers of venom proteins, enhancing their stability, bioavailability, and targeted antimicrobial effectiveness. This process is an improvement over the limitations associated with conventional antibiotic treatments. Specifically, the review emphasizes how SVEVs may provide a more targeted and safer alternative to synthetic carriers, with the additional potential for reduced toxicity and increased efficacy. But even with these promising results, the review acknowledges key challenges, including the need for standardized purification methods, rigorous safety evaluations, and scaling up clinical trials. The future of snake venom-based therapeutics lies in their optimization by methods (such as PEGylation, liposomal entrapment, and peptide modification) that would further expand the therapeutic applications of venom proteins. This review contributes to the existing literature by integrating existing knowledge regarding the antimicrobial potential of snake venom and imparting useful information regarding the development of venom-borne molecules as efficient therapies against drug-resistant bacterial infections. In the coming times, diligent research on the molecular pathways, therapeutic applications, and refinement of venom-based therapies will be crucial in realizing their complete therapeutic capacity. Rigorous preclinical and clinical studies are essential to validate safety and efficacy, while scalable manufacturing and regulatory frameworks will facilitate clinical translation. Additionally, AI-driven drug discovery and sustainable venom sourcing should be explored to ensure long-term viability. With continued innovation, snake venom-derived antimicrobials could emerge as a powerful tool in the fight against antibiotic resistance.

## Figures and Tables

**Table 1:** The key information about the venom components, their antibacterial targets, mechanisms, examples, and MICs against various bacteria.

Snake Venom Component	Type/Structure	Bacterial Targets	Mechanisms of Action	Example(s)	MIC (µg/mL)
**Metalloproteases (MPs)**	Protease enzymes (P-I, P-II, P-III)	Gram-positive and Gram-negative bacteria	Disrupt bacterial membranes, inhibit ion channels, hydrolyze structural components	Agkistrodon halys metalloproteinase (AHM), *Bothriopsis oligolepis* PIII-MP	1.875–60 (AHM), MIC for *S. aureus*: 80
**Serine Proteases (SPs)**	Single-chain enzymes (26–33 kDa)	Gram-positive bacteria	Membrane disruption, proteolytic degradation of proteins	*B. oligolepis* SP	80 (*S. aureus*)
**Phospholipase A2s (PLA_2_s)**	Enzyme hydrolyzing phospholipids	Gram-positive and Gram-negative bacteria	Membrane disruption via enzymatic hydrolysis	*Daboia russelii* PLA_2_, *Naja naja* PLA_2_	~11.1–20 (*S. aureus*), 10–20 (*Bacillus subtilis*)
**Three-Finger Toxins (3FTxs)**	Non-enzymatic proteins	Gram-positive bacteria	Disrupt bacterial membranes	A5 from *Naja naja*	No activity against Gram-negative bacteria
**Cysteine-Rich Secretory Proteins (CRISPs)**	Single-chain proteins (20–30 kDa)	Gram-negative bacteria, fungi	Modulate ion channels, disrupt bacterial membrane	Patagonin-CRISP (*Philodryas patagoniensis*)	15–7.5 *(P. aeruginosa*)
**L-Amino Acid Oxidases (LAAOs)**	Flavoproteins, catalyze oxidative deamination	Gram-positive and Gram-negative bacteria	Produce H_2_O_2_, leading to oxidative stress, biofilm inhibition	*Ophiophagus hannah* LAAO, *Trimeresurus jerdonii* LAAO	4.5–36 (*S. aureus*), 9–288 (varied bacteria)
**Antimicrobial Peptides (AMPs)**	Small cationic peptides	Gram-positive and Gram-negative bacteria	Disrupt bacterial membranes, inhibit cell wall synthesis	Cathelicidins, defensins, aprotinin	10–100 (*S. aureus, E. coli*)
**Nucleotidases**	Enzyme hydrolyzing nucleotides	Gram-negative bacteria	Degrade bacterial DNA and RNA, disrupt bacterial integrity	Nucleotidase from *Bothrops asper*	20–80 (*P. aeruginosa*)
**Snake Venom Phosphatases**	Enzyme (phosphatase activity)	Gram-negative bacteria	Disrupt bacterial surface structures, interfere with signal transduction	Phosphatase from *Naja naja*	50 (*E. coli*)

**Table 2:** A summary of the roles and therapeutic potential of snake venom-derived extracellular vesicles (SVEVs) in antibacterial drug delivery.

Aspect	Details
**Source and Nature of SVEVs**	Naturally secreted by snake venom gland epithelial cells. Nano-sized, lipid bilayer-enclosed vesicles.
**Bioactive Cargo**	AMPs (cathelicidins, defensins), enzymes (PLA2s, LAAOs, 5′-nucleotidases), toxins, lipids.
**Isolation Techniques**	Ultracentrifugation, SEC, EVTRAP, polymer precipitation, antibody-based capture.
**Mechanisms of Antibacterial Action**	Membrane damage, oxidative stress induction, enzymatic degradation of bacterial components.
**Advantages over Traditional Antibiotics**	Reduced resistance development, enhanced stability, bioavailability, and synergistic potency.
**Therapeutic Applications**	Wound healing, sepsis treatment, adjuvant to antibiotics, and biofilm penetration.
**Engineering for Drug Delivery**	Functionalization with targeting ligands/antibodies, encapsulation of antibacterial drugs like cathelicidins and defensins.
**Benefits as Delivery Vehicles**	Protection of cargo, targeted delivery, low immunogenicity, biocompatibility, and sustained release.
**Challenges to Clinical Translation**	Standardization of isolation, cytotoxicity removal, in vivo validation, and clinical testing.
**Notable Example**	Bothrops jararaca SVEVs delivering antimicrobial enzymes and proteins.
**Complementary Delivery Approaches**	Exosome-based delivery, PEGylation, liposomes, hydrogels, microneedle patches, and biopolymer films.

**Table 3:** Possible strategies for enhancing antibacterial applications of snake venom-derived components.

Delivery System	Advantages	Applications	References
**Polymeric Nanoparticles (e.g., Chitosan)**	Biocompatible, mucoadhesive, intrinsic antimicrobial activity, enhances venom stability, controlled release	Targeted antibacterial therapy, treatment of infections, reduced inflammation	[165,169,170,171]
**PEGylation of Snake Venom Toxins**	Improved pharmacokinetics, reduced immunogenicity, increased enzymatic activity, stability, reduced renal clearance	Development of new antimicrobial drugs, enhanced bioavailability, reduced toxicity	[138,139,140,141,142]
**Liposomes**	Enhanced immunogenicity, minimized toxicity, high encapsulation efficiency, targeted delivery	Snake venom-based immunization, antimicrobial therapy, drug resistance treatment	[143,144,145,146,147,148,149]
**Hydrogels**	Sustained release, local delivery, multifunctional (antimicrobial, hemostatic, regenerative properties)	Wound healing, infection control, tissue regeneration	[150,151,152,153,154,155]
**Microneedles**	Minimally invasive, controlled local delivery, reduced systemic toxicity, high therapeutic efficacy	Transdermal drug delivery, biofilm-associated infection treatment	[156,157,158,159,160,161]
**Biopolymer Films**	Stable antimicrobial action, biodegradable, functionalized for broad-spectrum activity	Infection-resistant wound dressings, antimicrobial medical devices, food packaging	[161,162,163,164,165]

**Table 4:** Strategies for optimizing snake venom-derived peptides for antibacterial applications and reducing toxicity.

Category	Strategy	Description/Example
1. Enhancing Antibacterial Activity	Amino Acid Substitution	Alters hydrophobicity, amphipathicity, and charge to enhance membrane interaction and disruption.
	Cysteine to Alanine Substitution	Prevents disulfide bond formation while retaining activity (e.g., crotamine, bothropstins).
	Tryptophan Incorporation	Enhances membrane penetration and destabilization (e.g., melittin, vipericidins).
	Positive Charge Addition	Boosts electrostatic interaction with bacterial membranes, especially effective against Gram-positive strains.
	Insertion/Deletion Mutations	Alters α-helix or β-sheet formation, affecting flexibility and membrane penetration.
2. Synthetic Approaches and Analog Design	Shortened Derivatives	Examples: LZ1, ZY13—retain potency, with improved pharmacokinetics.
	Directed Evolution	Introduces random mutations to improve affinity, activity, and proteolytic resistance.
	Synthetic Analogs	Engineered for better selectivity, in vivo stability, and broader activity profiles.
3. Reducing Venom Toxicity	Component Isolation and Modification	Focus on non-toxic components like BPPs; enabled drugs like Captopril.
	Genetic/Chemical Modification	Modify toxic molecules to preserve function but reduce systemic toxicity.
	Natural Inhibitors (e.g., sera, plant extracts, EDTA)	Neutralize enzymatic activity of venom components like PLA_2_, metalloproteinases.
	Monoclonal Antibodies (mAbs)	Target specific venom toxins with high specificity and low immunogenicity.
	Nanoparticle Delivery Systems	Enable targeted delivery and reduced off-target toxicity, potential for theranostic use.
	Combination Therapies	Use of mAbs, nanoparticles, or natural inhibitors alongside antivenoms.
	Immunization and Vaccine Development	Inactivated venom components used to stimulate protective immunity.

## Data Availability

There are no data to support the findings of this review.
